# Comparative and evolutionary analysis of chloroplast genomes from five rare *Styrax* species

**DOI:** 10.1186/s12864-025-11629-3

**Published:** 2025-05-07

**Authors:** Hao-Zhi Zheng, Guo-Xing Peng, Liao-Cheng Zhao, Wei Dai, Meng-Han Xu, Xiao-Gang Xu, Ming Tang

**Affiliations:** 1 Co-Innovation Center for Sustainable Forestry in Southern China, College of Life Science, Nanjing Forestry University, Nanjing, 210037 China; 2 College of Forestry, Jiangxi Agricultural University, Nanchang, 330045 China; 3 Laboratory of Systematic Evolution and Biogeography of Woody Plants, School of Ecology and Nature Conservation, Beijing Forestry University, Beijing, 100083 China; 4 Jiangxi Provincial Key Laboratory of Conservation Biology, Jiangxi Agricultural University, Nanchang, 330045 China

**Keywords:** *Styrax*; Chloroplast genome diversity, Adaptive evolution, Selection pressure, IR region expansion and contraction, Phylogenetic conflict

## Abstract

**Background:**

*Styrax*, a vital raw material for shipbuilding, construction, perfumes, and drugs, represents the largest and most diverse genus in the Styracaceae. However, there is a relative scarcity of research on *Styrax*, particularly in evolution and genetics. Therefore, this study conducted comparative and evolutionary analyses of the chloroplast genomes of five rare *Styrax* species (*S. argentifolius*, *S. buchananii*, *S. chrysocarpus*, *S. finlaysonianus*, and *S. rhytidocarpus*).

**Results:**

The results indicated that, despite high levels of conservation in chloroplast genome structure among these species, specific mutation hotspot regions exist, particularly involving the expansion and contraction of the IR region. Additionally, evidence of positive selection was detected in eight genes (*atpB*, *ccsA*, *ndhD*, *petA*, *rbcL*, *rpoC1*, *ycf1*, and *ycf2*), which may be associated with adaptive evolution in response to environmental changes. Phylogenetic analysis revealed conflicts between trees constructed based on coding sequences and complete chloroplast genomes for several species, which were similar to previous phylogenetic studies.

**Conclusion:**

This study underscores the importance of increasing sample sizes to enhance the accuracy of phylogenetic analyses and provides a new perspective on understanding the adaptive evolution of *Styrax* species. These findings are not only important for the conservation and sustainable use of *Styrax*, but also provide valuable insights for research in plant evolution and ecology within the genus.

**Supplementary Information:**

The online version contains supplementary material available at 10.1186/s12864-025-11629-3.

## Background

The chloroplast in green plants plays a vital role in the process of photosynthesis and energy provision [1], as well as actively participating in the biosynthesis of amino acids, fatty acids, vitamins, and pigments [2]. The structure of chloroplast genomes is characterized by a circular quadripartite organization, which includes two inverted repeat regions (IRs), a small single copy (SSC) region, and a large single copy (LSC) region that is greatly conserved [3]. In addition, chloroplast genes exhibit a relatively high degree of conservative and play a crucial role in essential biological processes such as photosynthesis, transcription, and translation [4]. The length of these sequences varies among different species, typically ranging from 120 to 160 kb [5]. Notably, this includes two sets of four ribosomal RNA genes and 30 tRNA genes that possess the remarkable ability to interact with all mRNA codons through subtle conformational adjustments [6, 7]. In angiosperms, the maternal inheritance of the chloroplast genome helps maintain the stability of species evolution [8]. However, the occurrence of mutational events provides valuable information for evolutionary research [9], population classification [10, 11], and serves as effective genetic markers to unravel intricate evolutionary processes [12]. Consequently, chloroplast genes present an ideal research subject for investigating species evolution [13].

The diversity of chloroplast genomes provides an abundant source of specific markers for elucidating phylogenetic relationships at various levels [14–16]. Additionally, due to the maternal inheritance of chloroplasts in most angiosperms [17], their divergence from nuclear phylogenetic relationships can offer valuable insights into speciation processes such as hybridization and incomplete lineage sorting [18, 19]. Therefore, the comparative analysis of chloroplast genomes holds significant potential for investigating plant evolution. The modification of gene content in the chloroplast genome can facilitate species’ adaptation to specific habitats and life strategies [20, 21]. Environmental changes and variations in habitats may exert selective genetic pressure, thereby leaving a trace of natural selection in genes associated with environmental adaptation. Genes that undergo positive selection typically enhance individual fitness and reproductive capacity [22]. Consequently, the identification of selection pressure and adaptive evolution of genes has emerged as a prominent research area in molecular, forming the foundation for germplasm resource studies.

As a particularly important genus, *Styrax* L. encompasses approximately 130 species and represents the most diverse, extensively distributed, and highly distinct category within Styracaceae [23]. Ser. *Cyrta* in *Styrax*, for instance, is found in temperate lowlands and tropical montane forests in eastern and southeastern Asia as well as North America. The average annual precipitation ranges from 80 to 300 cm with no prolonged dry season [24, 25]. Due to its aromatic properties and wood characteristics, *Styrax* can be utilized as a raw material for shipbuilding, construction, perfume, cosmetics, and pharmaceuticals [25, 26]. Furthermore, the seed oil or resin of several *Styrax* species is a valuable medicinal ingredient and raw material for the manufacture of aromatic oils [27].

The classification of *Styrax* based on morphological characteristics has been a subject of considerable controversy throughout history, largely due to the significant impact of environmental factors on the morphological changes observed in the various species. At present, the majority of research on *Styrax* is centered on the sequencing, assembly, and construction of phylogenetic relationships of chloroplast genomes, most of which pertain to individual species [28–31]. Fritsch conducted a morphological phylogenetic analysis of *Styrax* and made revisions to the taxonomy of interspecific relationships within the genus [32]. He divided *Styrax* into Sect. *Styrax* (ca. 33 species) and Sect. *Valvatae* (ca. 97 species), while also dividing Sect. *Styrax* into Ser. *Styrax* and Ser. *Cyrta*, Sect. *Valvatae* is divided into Ser. *Valvatae* and Ser. *Benzoin*. By utilizing several chloroplast sequences (*ndhF*-*rpl32*-*trnL*, *trnL*-*trnF*, *trnS*-*trnG*, and *trnV*-*ndhC*) as well as nrDNA internal transcribed spacer (ITS), Fritsch and Morton successfully establish a robust phylogenetic relationship that supports *Styrax* as a monophyletic group [24]. Furthermore, they classify the South American dioecious branch into two distinct sublines: *Styrax* subseries *Latifoli* and *Foveolaria* [33]. Additionally, Song et al. employ DNA polymorphism analysis to identify *ycf1*b and *trnT*-*trnL* as specific DNA barcodes for *Styrax* in order to elucidate intergeneric and interspecific relationships with greater precision [5]. The currently available samples of chloroplast genomes only represent a quarter of the entire genus, and there is a lack of research on rare species. Debates among scholars regarding the synonymization and segregation of species names have been ongoing in the study of taxonomy and systematics [25, 34]. For example, *S. japonicus* has a wide distribution and diverse morphological characteristics, making it one of the most heterogeneous species in *Styrax*. Although *S. grandiflorus* is currently used as a synonym for *S. japonicus*, phylogenetic analysis of the chloroplast genome suggests that *S. japonicus* and *S. grandiflorus* are positioned in separate branches and exhibit distinct morphologies from each other [5, 28].

While current research has offered some insights into *Styrax*, the understanding of its adaptive evolution remains limited in the field. Therefore, it is crucial to further investigate the adaptive evolution and diversity of its chloroplast genome to enhance our comprehension. As a result, in this study, we conducted sequencing and analysis on five *Styrax* species while incorporating additional data from NCBI for a comprehensive examination. Specifically, our objectives are to (1) analyze the distinct characteristics of *Styrax* and genes associated with adaptive evolution, (2) reconstruct and compare phylogenetic relationships, and (3) explore potential patterns of adaptive evolution in *Styrax* by examining its associated chloroplast genes and phylogeny.

## Result

### Assembly and annotation of the *Styrax* chloroplast genome

In this study, the assembly of five chloroplast genomes revealed a typical quadripartite structure, consisting of one LSC region, one SSC region, and a pair of IR regions. The genome sizes ranged from 157,817 bp to 158,015 bp with GC content ranging from 36.9 to 37% (Supplement Table 1). Subsequently, it was observed that the five species each contained eight rRNA, 37 tRNA, and 87 protein-coding genes. Additionally, the *clpP*, *rps12*, and *ycf3* gene harbors two introns. All genes can be categorized into three types: (1) Photosynthesis; (2) self-replication; and (3) others (Supplement Table 2).

### Analysis of the Chloroplast genome structure of *Styrax*

**Fig. 1:**
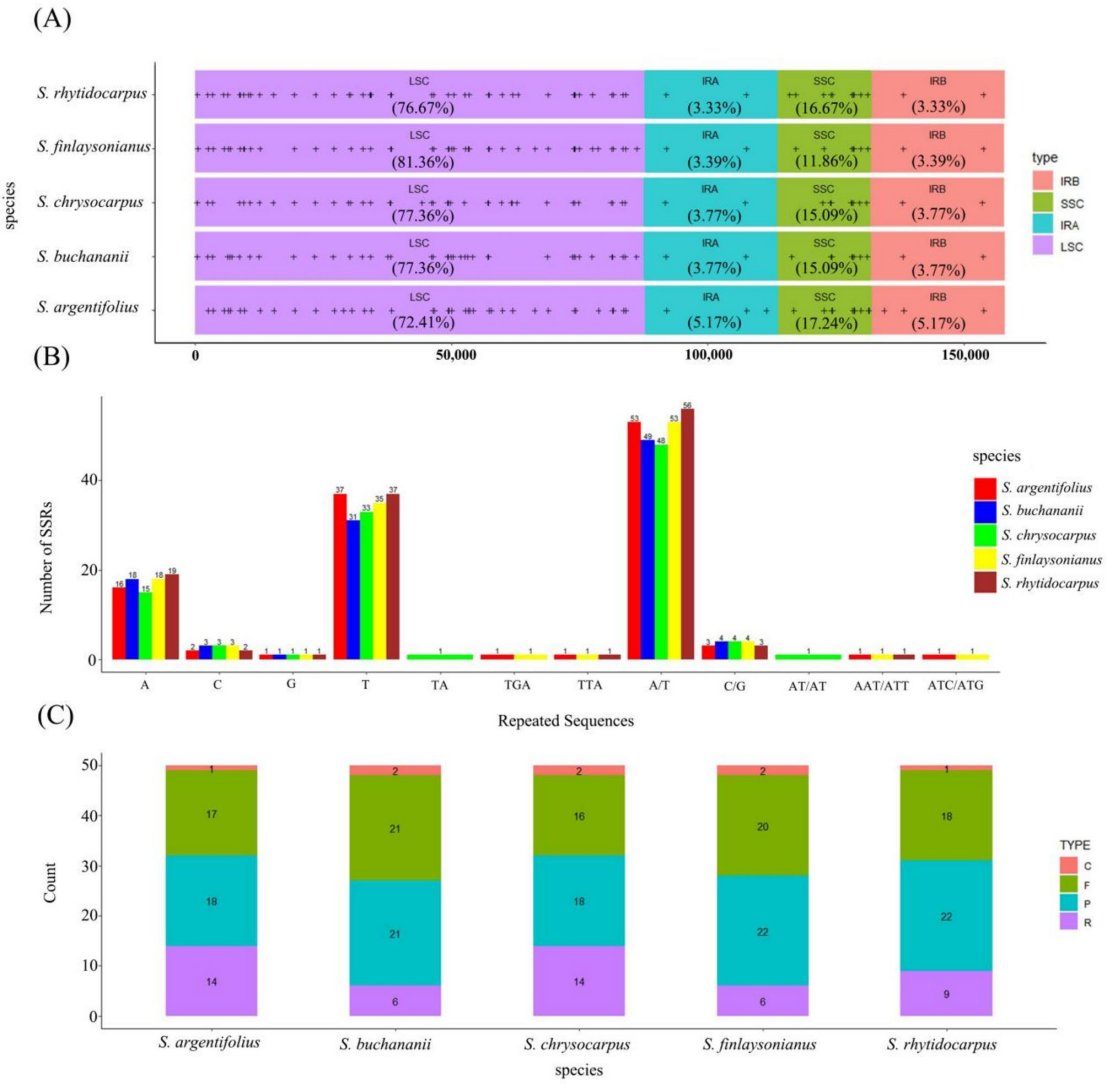
Analysis of SSR sites and repetitive sequences in five chloroplast genomes. (**A**). Distribution of SSRs in the five samples; (**B**). Number of different SSRs loci types; (**C**). Number of different repeat types. Note: In (**A**), symbol (+) represented the position of SSRs, and the proportion of text displayed; In (**C**), C: complementary repeats, F: forward repeats, P: palindromic repeats, R: reverse repeats

By utilizing OGDRAW for the visualization of chloroplast genomes among multiple species, it was observed that there existed a remarkable similarity in genome structure across various *Styrax* species (Supplement Fig. 1). Through SSR analysis, the *Styrax* chloroplast genome was found to contain a total of SSRs, with the majority (72.41–81.36%) located in the LSC region. The IR regions contained between 3.33 and 5.17% of SSR loci, while the SSC region included between 11.86 and 17.24% (Fig. 1A). The statistical analysis of SSR markers across five species highlighted that the A/T repeat type was particularly abundant, with all five species exhibiting a relatively high number of SSR markers in this category. Notably, *S. argentifolius*, *S. finlaysonianus*, and *S. rhytidocarpus* each possessed one SSR marker for the AAT/ATT repeat type. Additionally, both *S. argentifolius* and *S. finlaysonianus* were found to have one SSR marker for the ATC/ATG repeat type. In contrast, *S. chrysocarpus* uniquely exhibited one SSR marker for the AT/AT repeat type (Fig. 1B). There were 50 repeats in these five species, which included complementary, forward, palindromic, and reverse repeats. In general, the proportion of palindromic sequences is highest among the five species. The number of repetitive types in *S. argentifolius* and *S. chrysocarpus* is similar, while that in *S. buchananii*, *S. finlaysonianus* and *S. rhytidocarpus* is comparable. (Fig. 1C).

**Fig. 2:**
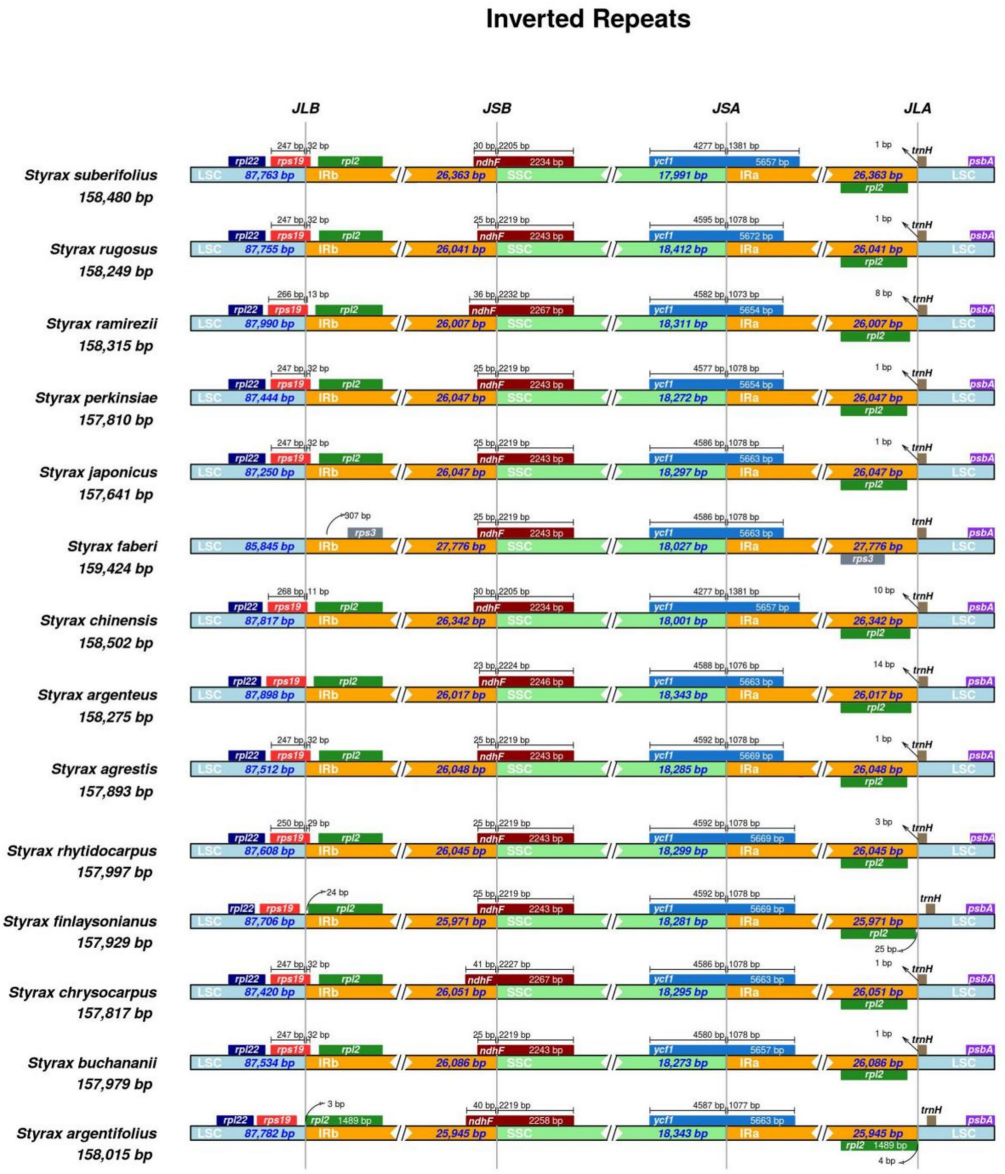
Comparison of chloroplast genome structure in five species and five closely related species. IR (Inverted repeat), LSC (Large single copy) and SSC (Small single copy) regions and border genes are indicated. Note: JLA: junction between LSC and IRa; JLB: junction between LSC and IRb; JSA: junction between SSC and IRa; JSB: junction between SSC and IRb

The complete chloroplast genome of *Styrax* was analyzed using the reference chloroplast genome of *S. japonicus* in mVISTA (Supplement Fig. 1), revealing a significant level of similarity and conservation among the chloroplast genomes within the genus *Styrax*. Notably, coding regions exhibited higher levels of conservation compared to non-coding regions, while the IR regions demonstrated lower variability than the LSC and SSC regions. Additionally, the IR region of these five species exhibited varying degrees of expansion and contraction when compared to other closely related species (Fig. 2). The *rps19* was located at LSC-IRB junction, *ndhF* and *ycf1* were located at the SSC-IR junction, *trnH* was located at IRA-LSC junction. Notably, *rpl2* was found to be significantly closer to the IR-LSC boundary within *S. argentifolius* and *S. finlaysonianus*, and *trnH* was found to have shifted entirely into the LSC region due to the contraction of the IR region.

### Codon bias analysis and selective pressures in the evolution

**Fig. 3:**
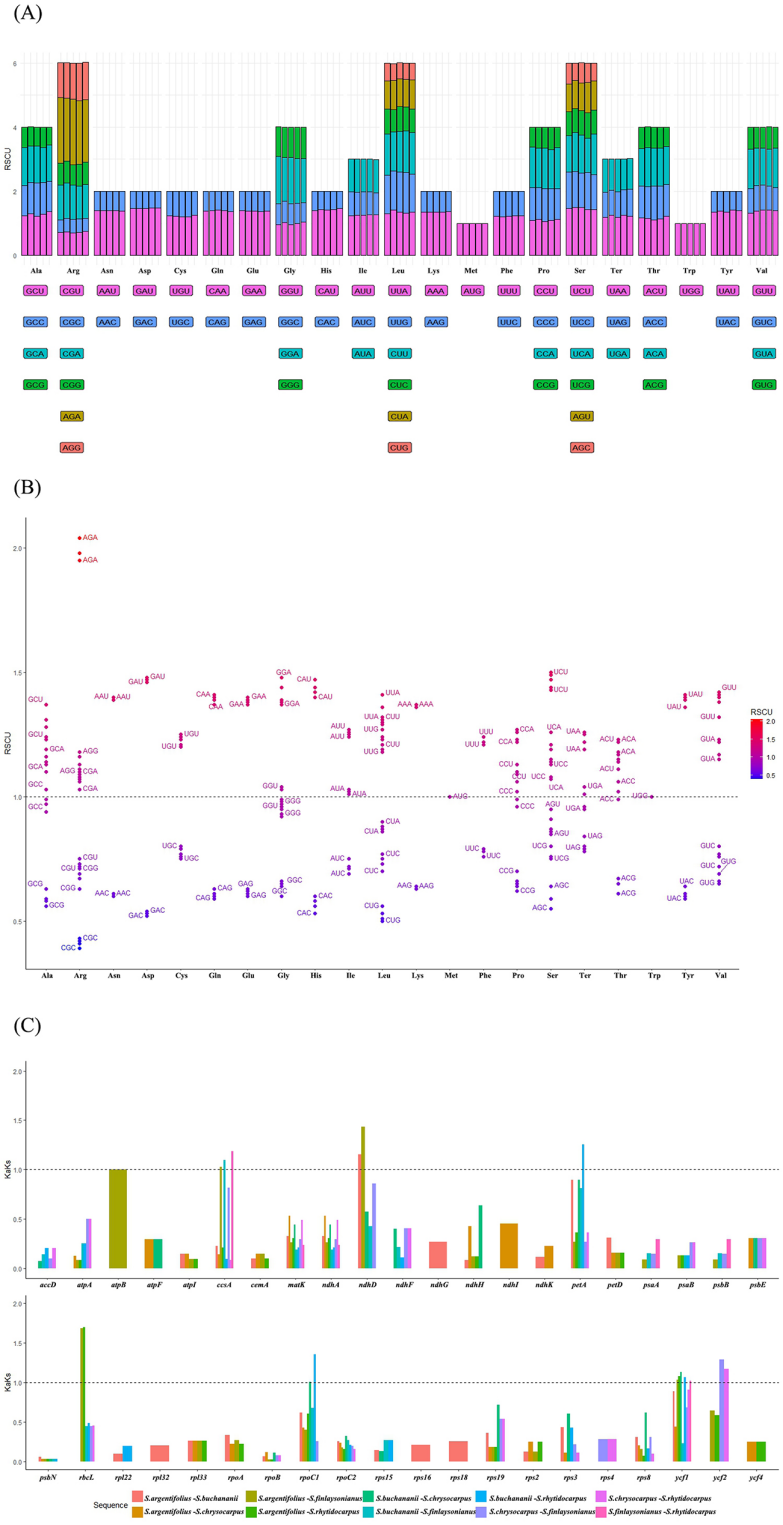
Relative synonymous codon usage and selective pressures in the evolution. (**A**). Codon content of 20 amino acids and stop codons in all protein-coding genes of five chloroplast genome; (**B**). Distribution of codon preference in five species; (**C**). Ka/Ks values of protein-coding genes of the five comparative combinations. Note: in (**A**), the top panels show the RSCU for the corresponding amino acids, and the colored blocks shown below represent different codons; In (**C**). Ka: nonsynonymous; Ks: synonymous

The examination of five chloroplast genomes demonstrated that the GC and GC3s composition within the codons was consistently lower than 0.5, suggesting a predilection towards A/T bases and A/T-ending codons in *Styrax* chloroplast genomes. The synonymous codon usage (RSCU) values showed similarity across the five *Styrax* chloroplast genomes (Fig. 3A). A total of 37 codons exhibited an RSCU value greater than 1 (Fig. 3B), with only one of these codons ending in G (UUG). Among the codons with an RSCU value less than 1, except for UGA and CUA, which terminate in A, the remaining codons conclude with either C or G.

Since some genes had Ks values of 0, resulting in an invalid Ka/Ks ratio. Of the 80 common genes, only 42 genes were included in the Ka/Ks analysis (Fig. 3C). The result suggested that 8 genes (*atpB*, *ccsA*, *ndhD*, *petA*, *rbcL*, *rpoC1*, *ycf1*, and *ycf2*) possessed Ka/Ks ratios > 1 in at least one pairwise comparison among the five species.

### Nucleic acid polymorphism analysis

**Fig. 4:**
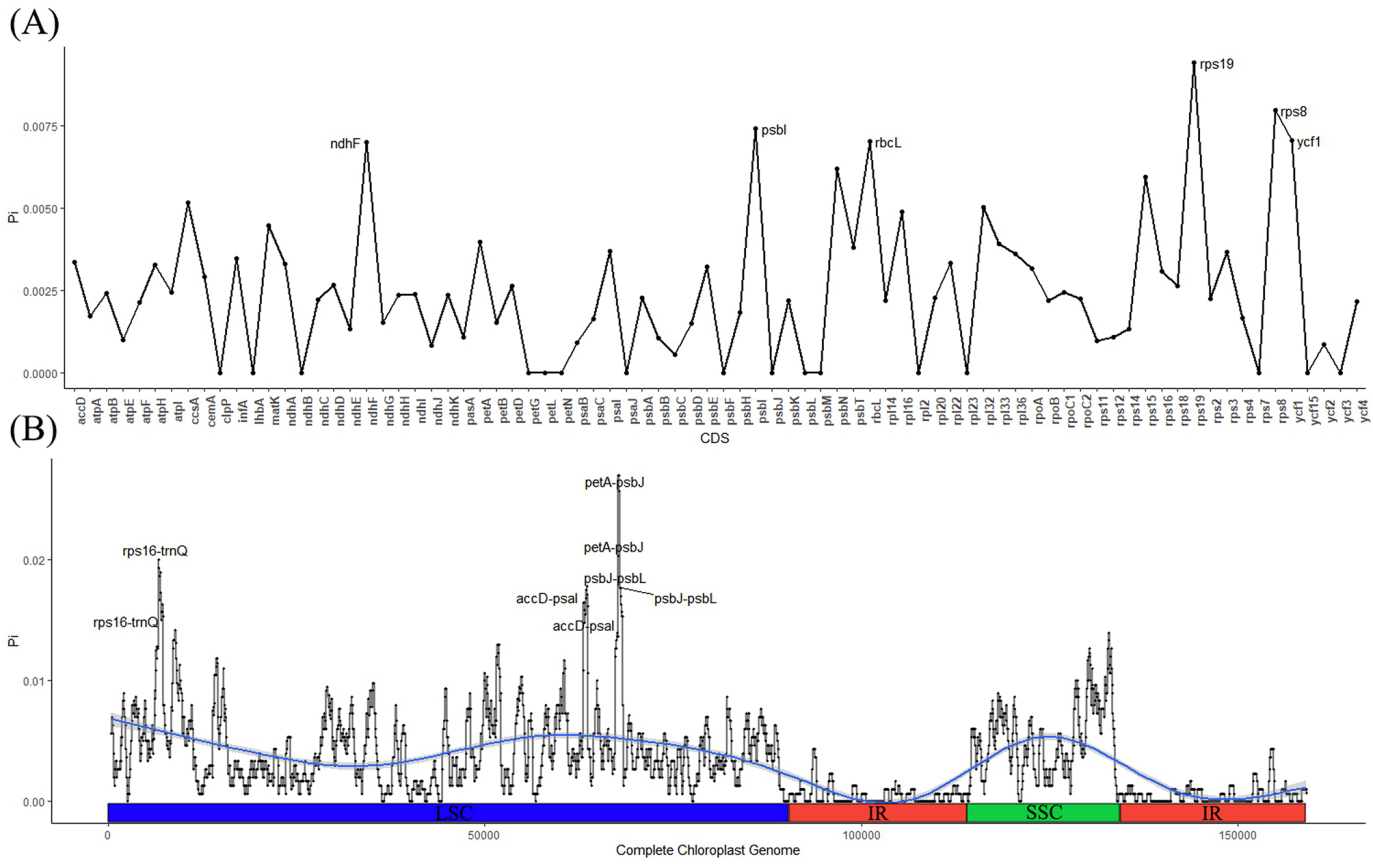
Nucleotide diversity of chloroplast genomes in five *Styrax* species. (**A**). Pi in CDS; (**B**). chloroplast genome Pi value. Note: window length: 600 bp, step length: 50 bp; X axis: position of the midpoint of each window; Y axis: Pi of each window. The blue line represents the trajectory of the value of Pi

In the analysis of Pi value in protein-coding sequences (Fig. 4A) and complete chloroplast genome sequences (Fig. 4B), the findings revealed varying levels of divergence across these three databases. The Pi values of *ndhF*, *psbI*, *rbcL*, *rps8*, *rps19*, and *ycf1* among the 80 protein-coding sequences were observed to exceed 0.07. In intergenic regions, four fragments (*accD-psaI*, *petA-psbJ*, *psbJ-psbL*, *rps16-trnQ*) were detected with high Pi values. Moreover, the analysis of the entire chloroplast genome revealed a higher level of conservation in the IR region compared to the SC region.

### Phylogenetic analysis

**Fig. 5:**
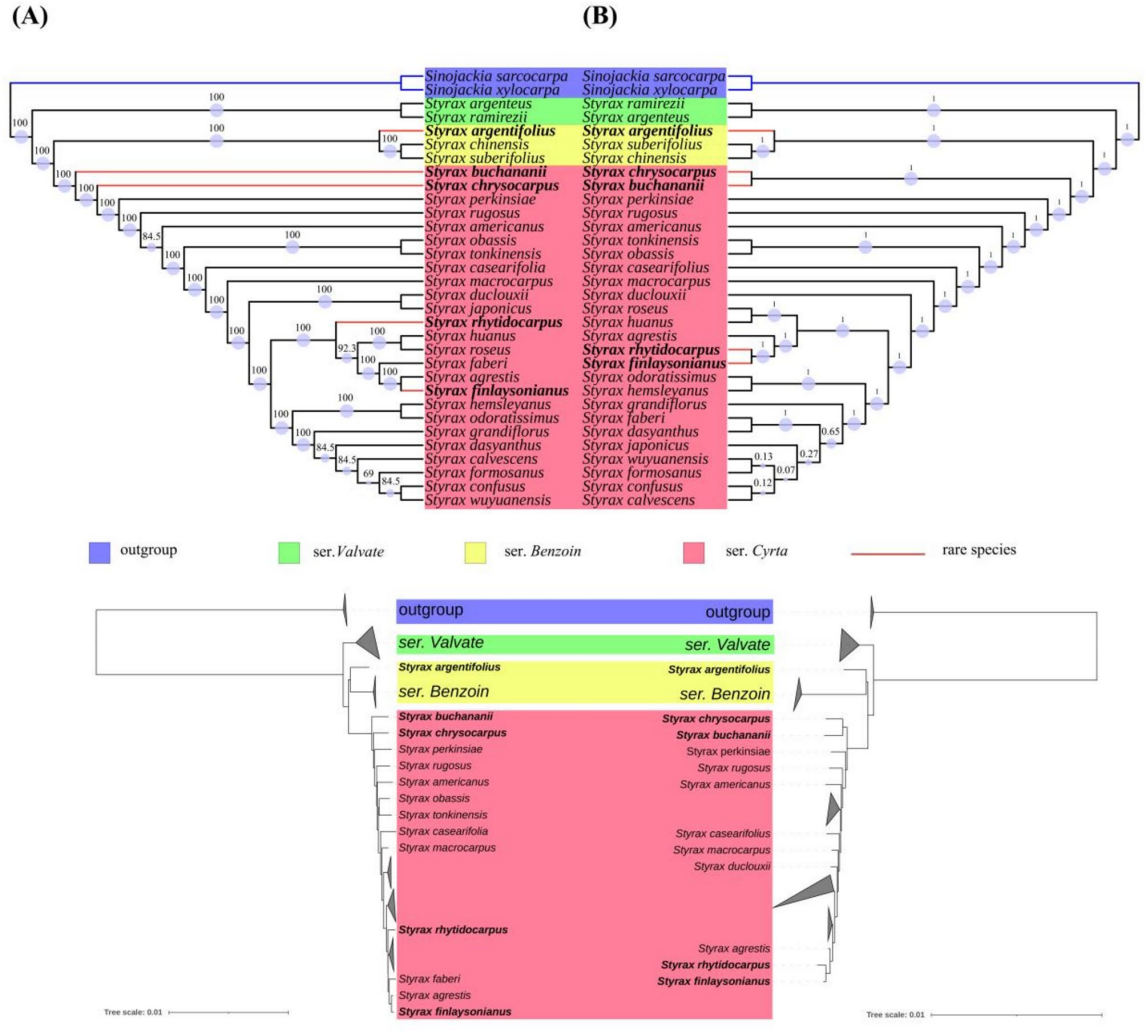
Phylogenetic tree analysis using Maximum Likelihood (ML) and Bayesian inference (BI) based on complete chloroplast genomes and CDS sequences. (**A**) Complete chloroplast genomes with ML method analysis. (**B**) CDS sequences with BI method analysis. Note: the blue circle at the branch node signifies the support rate of employing the bootstrap method, with its area directly proportional to the degree of support

ModelFinder determines that the optimal model for both ML and BI methods is GTR + G. The topology based on CDS sequences and complete chloroplast genomes shows similarity, with reduced internal branches in *Styrax* indicating low differentiation (Fig. 5).

In ML tree, *S. chrysocarpus* and *S. buchananii* each form individual clades, but formed a single clade in the CDS tree. Also, in ML, *S. finlaysonianus* and *S. agrestis* composed into a sub-branch and form a monophyletic group with *S. rhytidocarpus*, *S. hunans*, *S. roseus*, and *S. faberi*. However, in the CDS tree, it was observed that *S. finlaysonianus* and *S. rhytidocarpus* clustered into a subclade and formed a monophyletic group with *S. hunans*, *S. roseus*, and *S.agrestis.* Furthermore, the position of *S. argentifolius* did not change between the two methods. Moreover, apart from the five species mentioned in this study, there are instances of lower support rates for certain species in these two phylogenetic trees, such as *S. dasyanthus*, *S. calvescens*, and *S. confusus*, among others.

In general, the four remaining species, except for *S. argentifolius*, exhibited varying degrees of phylogenetic conflict in both analyses. This conflict was observed not only within these four species but also in *S. faberi*, *S. japonicus*, and *S. grandiflorus*.

## Discussion

In this study, we assembled chloroplast genomes of five rare *Styrax* species and furtherly performed comparative and evolution analyses. The results indicate that despite a high degree of similarity in their chloroplast genome structure, there are still discernible mutation hotspots. Specifically, (1)The results of the SSR analysis of the chloroplast genome indicated polymorphism; (2) the IR region of the chloroplasts in the five species exhibited varying degrees of expansion or contraction; (3) Evolutionary analysis revealed positive selection in eight genes; (4) Phylogenetic trees based on CDS and complete chloroplast genomes displayed multiple conflicts in relationship inference. Therefore, we will discuss the aforementioned four points.

### Fluctuations of SSR in *Styrax* chloroplast genome

As a highly polymorphic marker, SSR holds significant application value in plant taxonomy and germplasm identification [35]. Compared with other markers, SSR possesses the characteristics of high polymorphism and ease of detection, being particularly suitable for differentiating closely related species [36–38]. In chloroplast genomes, the number of SSR motifs varies even in the same genotype due to changes in tandem arrays of SSR motifs [39].

In this study, the majority of SSR were identified within the LSC region of the chloroplast genome, predominantly as single nucleotide repeats. Notably, Zheng et al. reported that SSR in the LSC region of *Styrax japonicus* Siebold & Zucc. accounted for 77.59–79.66% [40]. Utilizing the same MISA parameter settings, our analysis revealed that the proportion of SSR in the five rare species ranged from 72.41 to 81.36%, with similar fluctuations observed in the SSC and IR regions. Research conducted by Morgante et al. indicates that SSR locus tend to be more common in the non-repetitive DNA regions of plant genomes [41], which may explain the variability in SSR proportions within the LSC and SSC regions of the *Styrax* chloroplast genome. Furthermore, the locus of SSRs exhibits significant dynamic changes, particularly in intergenic and non-coding regions, reflecting both genomic evolution and functional regulation [42]. Previous studies have demonstrated that SSR polymorphism can influence an organism’s adaptability to environmental changes [43]. Therefore, we hypothesize that the fluctuation in SSR ratios across the LSC, SSC, and IR regions in our five species may represent an evolutionary adaptation of chloroplast genomes to diverse habitats, potentially contributing to the survival and proliferation of *Styrax* in varied ecosystems.

In summary, SSR analysis offers more comprehensive data support for comparative genomics research. Furthermore, the utilization of SSR markers in gene mapping and breeding programs is expected to expand significantly, particularly in marker-assisted selection and genome editing. Importantly, SSR analysis will facilitate a deeper understanding of genomic dynamics and its role in evolution and adaptation, thereby providing a scientific foundation for biodiversity conservation and sustainable use.

### Expansion and contraction of IR region

The findings of this study suggest that the boundary genes of five rare *Styrax* species appear to be consistently maintained with no significant alterations. However, it was observed that the *rps19* and *trnH* of *S. argentifolius* and *S. finialysonlanus* have fully encroached into the LSC region, indicating a contraction of the IR region. Furthermore, the length of the IR region in *S. buchananii* is larger than that of other species, while the LSC region is slightly shorter, reflecting an expansion of the IR region.

The expansion or contraction of IR regions in the chloroplast genome is typically attributed to various factors such as genome rearrangement [44], random mutations [45], evolutionary pressure [46], and gene transfer [47]. While the IR region is generally considered highly conserved [48], variations in boundary genes can result in gene loss or pseudogene formation, a common phenomenon in chloroplast genome evolution that significantly impacts its length [49].

The IR region of the chloroplast genome is generally considered to be the most stable, and its expansion can enhance the genetic stability of the entire genome [50]. Conversely, contraction of the IR region may lead to increased genetic diversity in the overall genome. Additionally, current studies have demonstrated that the expansion or contraction of the IR region influences gene dose, which subsequently affects gene expression. For instance, the IR region encompasses ribosomal RNA operons, and its expansion can raise the dose of rRNA genes, thereby enhancing the biosynthetic capacity of the ribosome and improving the expression efficiency of genes related to photosynthesis [48]. In tobacco, the removal of the IR region led to a slight reduction in the number of ribosomes, suggesting that the gene dosing effect of the IR region has a significant impact on ribosome biosynthesis [51].

Studies on the chloroplast genome of *Gentianinae* (Gentianaceae) indicate that the varying degrees of contraction and expansion of IR regions in multiple species of *Gentianinae* might be caused by gene loss and duplication. Meanwhile, the study revealed that microstructural changes in the genome and gene loss are associated with alterations in selection pressure [52, 53]. For instance, the loss of the ndh gene complex could be related to changes in environmental stress, such as variations in temperature, light, and moisture conditions. In some plants adapted to drought or bright light environments, the loss of ndh genes might be an adaptive evolution [54]. Thus, we hypothesize that changes in the IR region influence the function of the chloroplast genome to a certain extent, leading to adaptation to specific environmental climates to enhance the reproduction and regeneration of species.

In this study, both contraction and expansion were observed in the chloroplast genomes of five rare species at *Styrax*, indicating dynamic changes in their genomes. Such dynamic variations are often evolutionary responses to environmental pressures and adaptive evolution aimed at improving species’ ability to adapt. In summary, the expansion and contraction of the IR region have significant impacts on plant survival and reproduction by influencing gene dose, genome stability, and gene expression. These alterations not only assist plants in adapting to diverse environmental conditions but might also offer a selective advantage during evolution.

### Positive selection of eight genes

In this investigation, a total of eight genes (*atpB*, *ccsA*, *ndhD*, *petA*, *rbcL*, *rpoC1*, *ycf1*, and *ycf2*) were found to be under positive selection. This is an uncommon occurrence in other taxa where previous studies have only identified two or three positively selected genes [40, 55]. Therefore, the chloroplast genome characteristics observed in the five species studied indicate ongoing adaptive evolutionary processes within *Styrax*.

Firstly, the majority of these genes are directly involved in photosynthesis or closely related physiological processes. For instance, the *atpB* gene encodes a subunit of ATP synthase that is essential for photophosphorylation [56], while the *rbcL* gene encodes the large subunit of Rubisco, a key enzyme for CO_2_ fixation [57]. In the study of Polygonaceae, the *atpB* gene was identified as one of the genes undergoing positive selection [58]. These plants are widely distributed in northern temperate and tropical regions, often facing drought and high temperatures. The positive selection of *atpB* likely enhances ATP synthase activity and stability, enabling more efficient ATP synthesis under these stressful conditions and supporting photosynthesis [59]. Similarly, in *Ardisia*, which mostly grows in low-light forest understories, the *rbcL* gene was found to be positively selected [60]. This selection may optimize Rubisco activity and efficiency, improving photosynthesis in low-light environments.

The *ccsA* gene is involved in copper metabolism within chloroplasts, with copper serving as a cofactor for certain key enzymes in photosynthesis [61]. The *ndhD* gene is associated with the NADH dehydrogenase in chloroplasts and linked to the regulation of the photosynthetic electron transport chain [62, 63]. The protein encoded by the *petA* gene is an essential component of the photosynthetic electron transport chain [64, 65]. In studies of *Scutellaria*, the *ccsA* was found to be under positive selection, which may be related to the plant’s ability to adapt to environmental stresses such as drought and salinity [66]. Positive selection of this gene may enhance photosynthetic efficiency under adverse conditions. In Orchidaceae, the *ndhD* was found to be under positive selection in several species. This selection may optimize the stability of the photosynthetic electron transport chain, thereby improving photosynthetic efficiency under high light conditions [67]. In the genus *Quercus*, the *petA* gene was found to be under positive selection in some species. This selection may enhance the stability of the photosynthetic electron transport chain under drought conditions, thus improving photosynthetic efficiency [68].

The *rpoC1* gene encodes a DNA-dependent RNA polymerase, which is a key component of chloroplast gene expression [69]. The *ycf1* and *ycf2* are two larger open reading frames (ORFs) in the chloroplast genome, and the proteins they encode play important roles in plant physiological processes. The *ycf1* gene is essential for plant survival and is thought to be involved in the co-assembly and stability maintenance of photosynthesis complexes [70, 71], which are closely related to photosynthesis efficiency. In addition, limited research suggests that *ycf2* may be involved in the transport and quality control of proteins within the chloroplast, and is crucial for cell survival [72]. Although the functions of *ycf1* and *ycf2* genes are not fully understood, they may play a role in certain biological processes of the chloroplast [73]. In Polygonaceae plants, the *rpoC1* is under positive selection, likely enhancing chloroplast gene expression and photosynthetic efficiency in drought and high light conditions [58]. In Oenantheae, the *ycf1* and *ycf2* genes, crucial for plant survival and photosynthesis, show significant positive selection. *ycf1* may stabilize photosynthetic complexes, and *ycf2* may control protein quality in the chloroplast, aiding photosynthesis in low light and oxygen environments [74]. These genes have undergone positive selection, which may indicate that they play an important role in regulating chloroplast gene expression or responding to environmental changes.

Furthermore, these genes subject to positive selection may also be associated with the adaptation of plants to other abiotic environmental factors such as temperature, water availability, and salinity [75–77]. For instance, certain mutations could enhance the plant’s tolerance in drought or high-salt environments [78–80]. From an evolutionary standpoint, the positive selection of these genes reflects the long-term adaptation of plants to their environment. This process may involve speciation, ecological niche variations, and the generation of biodiversity [81–83].

Overall, the eight genes under positive selection can be categorized into three classes based on their functions. The positive selection of these genes may enhance the photosynthetic efficiency, optimize energy utilization, and improve environmental adaptability of the five rare species studied, thereby aiding the plants in better survival and reproduction in complex natural environments. Specifically, the positive selection of *atpB*, *rbcL*, and *petA* directly increases the efficiency of photosynthesis, enabling plants to perform photosynthesis more effectively under different light conditions. The positive selection of *ccsA* and *ndhD* optimizes the process of energy utilization, enhancing the stability and efficiency of photosynthesis. The positive selection of *ycf1* and *ycf2* improves the adaptability of plants under adverse conditions, strengthening their survival and reproductive capabilities. The combined action of the positive selection of these genes enables plants to better adapt and survive in complex natural environments. Through these studies, we can gain a better understanding of the molecular mechanisms of plant adaptive evolution and potentially provide new strategies for plant improvement and ecological conservation in an academic context.

### Phylogenetic analysis

The results of the phylogenetic analysis indicated that the relationships among *S. argentifolius*, *S. chrysocarpus*, and *S. buchananii* were stable in both the CDS-based and complete chloroplast genome-based trees. However, conflicts were observed in the relationship between *S. finlaysonianus* and *S. rhytidocarpus*. Despite these differences, the overall positions of each species were generally consistent with previous research [5, 40].

The conflicting relationships between evolutionary trees constructed from CDS sequences and complete chloroplast genomes pose a complex problem, potentially arising from multiple factors. Firstly, the two phylogenetic trees constructed are both maternally inherited. However, the reasons for the conflicts in phylogeny inferred from chloroplast genomes remain unclear. A similar situation has been observed in the genus *Gentiana* [84], where possible causes such as heterogeneous recombination in the plastid genome and a complex history of structural evolution have been proposed [16, 85, 86]. Although no studies have detected recombination in the *Styrax* chloroplast genome, the possibility of plastid recombination cannot be ruled out.

Moreover, hybridization is common causes of divergent topologies in evolutionary trees built from different genomic regions [87]. Hybridization events may result in genetic recombination [88]. For instance, during the evolutionary process of *Salix* and *Populus*, there were several ancient hybridization events that led to the movement of genes between different species, causing some species’ genomes to retain genes from other species. This resulted in inconsistencies among the constructed phylogenetic trees, thereby leading to phylogenetic conflict [89]. Additionally, genetic recombination and gene loss or pseudogenes may also confound systematic signals [90]. Genetic recombination within the chloroplast genome could lead to changes in gene order, while gene loss or the emergence of pseudogenes may impact sequence-based systematic analyses [91]. Furthermore, differential rates of molecular evolution and gene selection might also contribute to divergent trees based on various genomic regions. Some genes may exhibit varying evolutionary rates and patterns due to the influence of natural selection [92].

In addition to the five mentioned species, this study observed a phenomenon of low support for certain species. Given the whole scale of this genus, it is possible that only a portion of the true current systematic evolutionary relationships has been reconstructed. We speculate that the primary reason for this issue lies in the quantity of samples used to construct phylogenetic relationships. Firstly, an increase in sample size can significantly enhance the statistical power of phylogenetic analysis. More samples yield more genetic variation information, aiding in more accurate inference of evolutionary relationships between species and reducing uncertainty in tree topology [93]. Secondly, an increased sample size helps mitigate sampling bias and provides a more comprehensive view of genetic diversity, thereby improving tree resolution and making differentiation between closely related species or populations clearer [94]. Conversely, a reduction in sample size may lead to increased uncertainty in analysis results and raise the risk of misjudging evolutionary relationships [95]. For example, Moritz emphasized the importance of sample size in defining evolutionarily significant units within conservation biology. Insufficient samples may overlook important genetic variations and evolutionary signals, thus impacting our precise understanding of biological evolutionary history [96]. Additionally, Templeton et al. suggest that a decrease in sample size may obscure genetic structure within species, affecting our comprehension of both species formation and diversity [97].

In general, the currently available genetic data is insufficient to systematically depict the phylogenetic profile of *Styrax*. In the context of this study, the inclusion of five rare species has supplemented some nodes and branches. While this may seem insignificant for a genus with 130 members, we believe that further research and collection will provide new insights into *Styrax* and even Styracaceae.

## Conclusion

This study conducted sequencing and analysis of the chloroplast genomes of five rare *Styrax* species, revealing unique characteristics in terms of genome structure, adaptive evolution, and phylogenetic relationships. The main findings include four aspects: (1) Despite the high similarity in chloroplast genome structures among the five species, expansion and contraction of the IR region were observed, possibly reflecting dynamic genomic responses to chloroplast genome diversity within *Styrax*. (2) Signs of positive selection were detected in eight genes, a rarity in other lineages. These genes are directly involved in photosynthesis or related physiological processes, suggesting potential adaptation of *Styrax* species to diverse environmental conditions. (3) Conflict was observed among certain species in the phylogenetic tree constructed based on CDS sequences and complete chloroplast genomes, potentially attributed to factors such as gene flow, hybridization, genetic recombination, and gene loss. (4) This study underscores the importance of increasing sample sizes to enhance statistical power in phylogenetic analyses while reducing sampling bias and more comprehensively reflecting genetic diversity.

In conclusion, this study has not only advanced our understanding of the evolution of chloroplast genomes in *Styrax* species, but also laid a solid foundation for further research in evolutionary biology, ecology, and conservation biology. Considering the ecological and economic significance of *Styrax* species, the findings of this study hold important implications for the conservation and sustainable utilization of these species.

## Materials and methods

### Plant materials, genomic Dna isolation and genome sequencing

In this research, fresh blades of five species were collected from *S. argentifolius* (N 22.91, E 103.71)(Pingbian county, Yunnan province), *S. buchananii* (N 24.44, E 103.71)(Yijiang county, Yunnan province), *S. chrysocarpus* (N 23.42, E 103.71)(Malipo county, Yunnan province), *S. finlaysonianus* (N 21.92, E 101.25)(Mengla county, Yunnan province), and *S. rhytidocarpus* (N 24.82, E 112.67)(Lianzhou city, Guangdong province). Voucher specimens of five species, identified by Prof. Ming Tang, were preserved in the Herbarium of Jiangxi Agricultural University (JXAU) with the following IDs: Zrr 14, Zrr 34, Zrr 2, Zyq&Lyl 022, and Zwy 1905. Due to the ongoing construction of the website of this herbarium, the relevant information can be obtained by sending an email to the corresponding author Prof. Ming Tang or JXAU. All five species are native to China and exemplify natural distributions. The DNA from each sample was extracted following the protocol of the Plant Genomic DNA Kit (Beijing Quanshijin Biological Co., Ltd.). The concentration and purity of the DNA were evaluated using a Nanodrop 2000 instrument (Thermo Fisher Scientific, Waltham, Massachusetts, USA). The integrity of the DNA was evaluated using agarose gel electrophoresis. Following Illumina’s standard protocol, libraries were constructed from the extracted total DNA and sequenced on the Illumina NovaSeq 6000 platform (Illumina, Cambridge, MA, USA) with paired-end reads of 150 bp. All methods are exclusively conducted on *Styrax* plants for experimental purposes, strictly adhering to relevant institutional, national, and international guidelines and regulations.

### Chloroplast genome assembly, annotation and sequence analysis

The raw reads were filtered by CLC Genomics Workbench v9 and filtered sequences were assembled using the program SPAdes v3.13.1 [98]. The scaffolds were generated by connecting the resulting contig sequences using SSPACE v2.0 [99], followed by supplementation of the scaffolds with gap using Gapfiller v1.11 [100], the specific assembly process is placed in the Supplementary Materials.

To ensure the accuracy of chloroplast genome annotation, we initially conducted a Blast comparison of the assembled sequences with the NCBI database to identify the most suitable reference genome [101]. Subsequently, CPGAVAS2 and GeSeq were employed for chloroplast genome annotation [102, 103]. The resulting annotations were manually refined and corrected using Geneious v9.0.2 (Biomatters Ltd., Auckland, New Zealand), ultimately yielding precise annotation results.

The online tool REPuter is utilized to determine the size and location of forward, reverse, palindromic, and complementary repeats in the chloroplast genome [104]. Identification of Simple sequence Repeats (SSRs) using MISA v2.1(MIcroSAtellite Identification Tool) source code, including mono-, di-, tri-, tetra-, penta-, and hexa-nucleotides, minimum number (thresholds) were 10, 6, 5, 5, 5, and 5, respectively [105]. The chloroplast genome sequences were uploaded in GenBank (Accession Number: PQ276582-PQ276586).

In order to identify the inversion and rearrangement of chloroplast genomes, a comparison was conducted on 10 *Styrax* species (including five in this study) using the Mauve v2.3.1 plugin Geneious [106].

### Analysis of codon usage bias and selective pressures in the evolution

PhyloSuite v1.2.3 is employed to extract the full-length CDS sequence and concentration [107]. Subsequently, CodonW v1.4.2 is utilized for multiple analyses, including nucleotide compositions at the third position (A3s, U3s and G3s), GC content at third codon positions (GC3s), codon adaptation index (CAI), codon bias index (CBI), effective number of codons (ENC), and relative synonymous codon usage (RSCU).

To calculate the substitution rates of synonymous (Ks) and non-synonymous (Ka), as well as their ratios (Ka/Ks values), a total of 80 common protein coding sequences were extracted and aligned using MAFFT v7.526 [108]. The YN model, widely used in evolutionary studies, is capable of reflecting the evolutionary characteristics of sequences [109, 110]. Therefore, we utilized the YN algorithm in KaKs calculator v3.0 to compute the selection pressure and Ka/Ks values [111]. A Ka/Ks value less than 1 indicates negative selection (purification selection), while a value equal to 1 suggests neutral selection. A Ka/Ks value greater than 1 signifies positive selection (adaptive selection). It should be noted that in some cases, invalid results may occur when either Ka or Ks equals 0.

### Comparative analysis

The chloroplast genome typically consists of a large single copy region (LSC), a small single copy region (SSC), and a pair of invert repeat regions (IR). While the structure of the chloroplast remains relatively stable, variations in boundary genes and the length of each region reflect interspecific divergence and evolutionary characteristics to some extent. Therefore, IRscope was utilized for analyzing the five species and their sibling species in this study [112]. Additionally, the Shuffle-LAGAN model within mVISTA was employed to uncover genome differentiation and mutation hotspots [113]. DNAsp v6.12.03 was used for calculating nucleotide polymorphisms (Pi) in CDS sequences and complete chloroplast genomes [114], the analysis employed a step size of 50 bp with a window length of 600 bp.

### Phylogenetic analysis

Complete chloroplast genome samples from 32 *Styrax*, and 2 *Sinojackia* as outgroup were utilized as the database for phylogenetic analysis. The optimal model was determined using ModelFinder [115]. The complete chloroplast genome was then constructed with phylogenetic structures using the maximum likelihood method (ML) in IQ-tree v1.6.12 [116], with 1000 bootstrap replicates. Additionally, the CDS sequence using the Markov Chain Monte Carlo (MCMC) algorithm in MrBayes v3.2.7 [117], with 1,000,000 generations and sampling every 1,000 generations. The first 25% of trees from all runs were discarded as burn-in, and the remaining trees were used to create a majority-rule consensus tree through Bayesian inference (BI).

## Electronic supplementary material

Below is the link to the electronic supplementary material.

Supplementary Material 1

## Data Availability

The original contributions presented in this study are publicly available. These data can be found here: NCBI(Accession Number: PQ276582-PQ276586).
