# Supplementary material for: Comparative and evolutionary analysis of chloroplast genomes from five rare Styrax species
Source: BMC Genomics. 2025 May 7;26:450. doi: 10.1186/s12864-025-11629-3 (PMC12057227; doi:10.1186/s12864-025-11629-3)
Supplement: Supplementary file 1 — Supplementary Material 1 [file 12864_2025_11629_MOESM1_ESM.docx]

**Supplement Material**

**Filtering Criteria**

Fastp v0.20.0 (https://github.com/OpenGene/fastp) was used to filter the original data. The filtering criteria were as follows:

(1) Truncate the sequencing linker and primer sequence in the reads;

(2) Filter out the reads whose average quality value is < Q5;

(3) Filter out n reads whose number is > 5;

The high-quality reads obtained after the above series of quality control steps were the clean data.

**Assembly Process**

SPAdes v3.10.1 was used for genome assembly; kmer used 55, 87, and 121 respectively, and the assembly did not depend on the reference genome. However, due to the characteristics of second-generation sequencing, genomic repeats, genome-specific structure, and other reasons, the complete circular genome sequence could not be directly obtained by one-time splicing. Other strategies were used to obtain the complete circular genome sequence. The assembly process was divided into the following 7 steps:

Step 1: The seed sequence of the chloroplast genome was obtained by assembling the cpDNA sequences with SPAdes v3.10.1.

Step 2: kmer iterative extend seed if the result of step 2 was a contig, the result was determined to be a pseudo genome sequence, and step 6 was performed directly.

Step 3: Connect the contig sequence obtained in step 2 with SSPACE v2.0 [35] to obtain the scaffolds.

Step 4: Use Gapfiller v2.1.1 to make up the gaps for the scaffold sequences obtained in step 3.

Step 5: If a gap still existed after these operations, design primers, conduct PCR sequencing, and reassemble until the complete pseudo genome sequence was obtained.

Step 6: The sequencing reads were aligned to the pseudo genome to correct the genome.

Step 7: According to the structure of the chloroplast, the corrected pseudo genome was rearranged to obtain the complete chloroplast circular genome sequence.

**Supplement Figure**


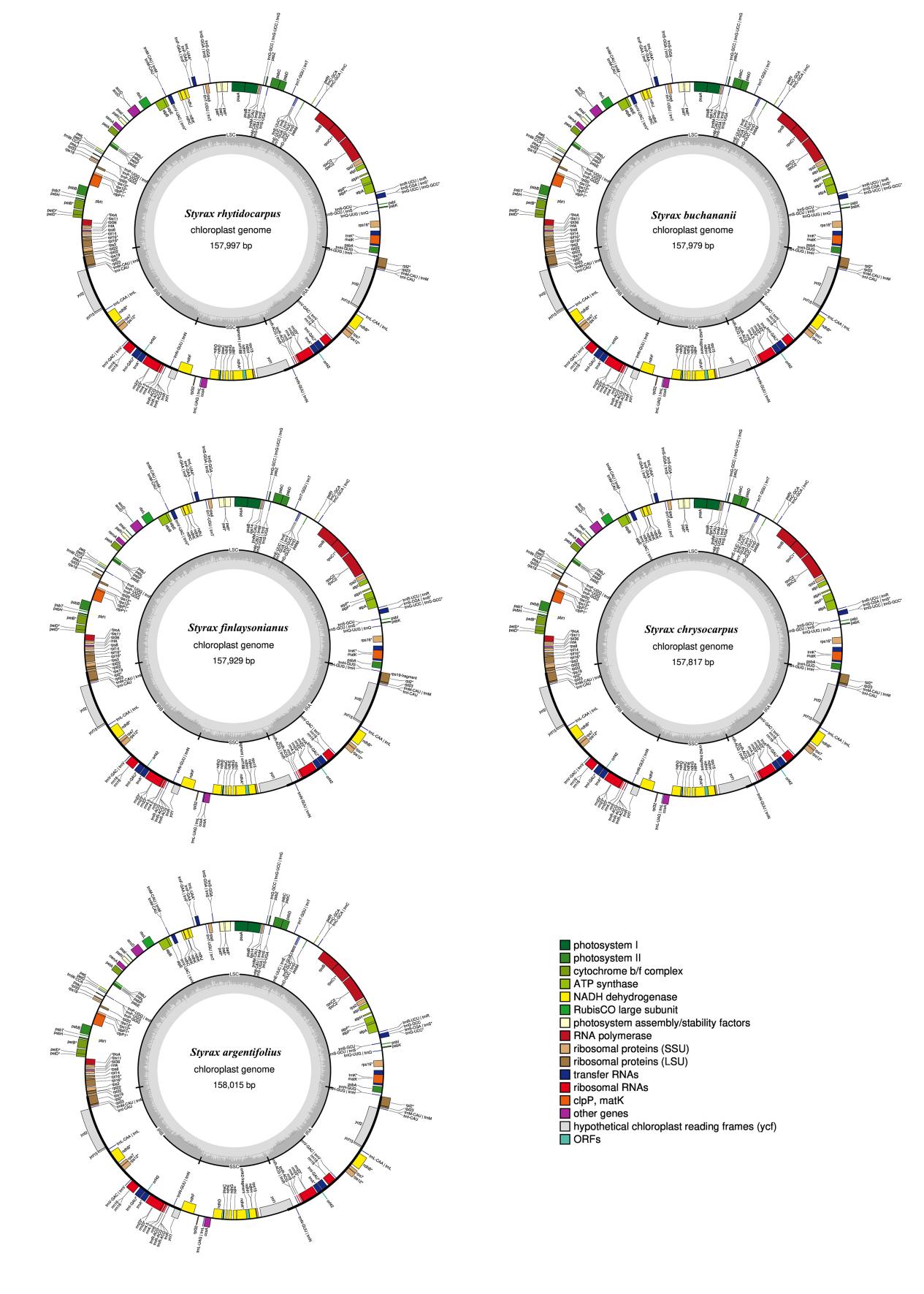


**Figure S1.** Gene map of the five *Styrax* chloroplast genomes.


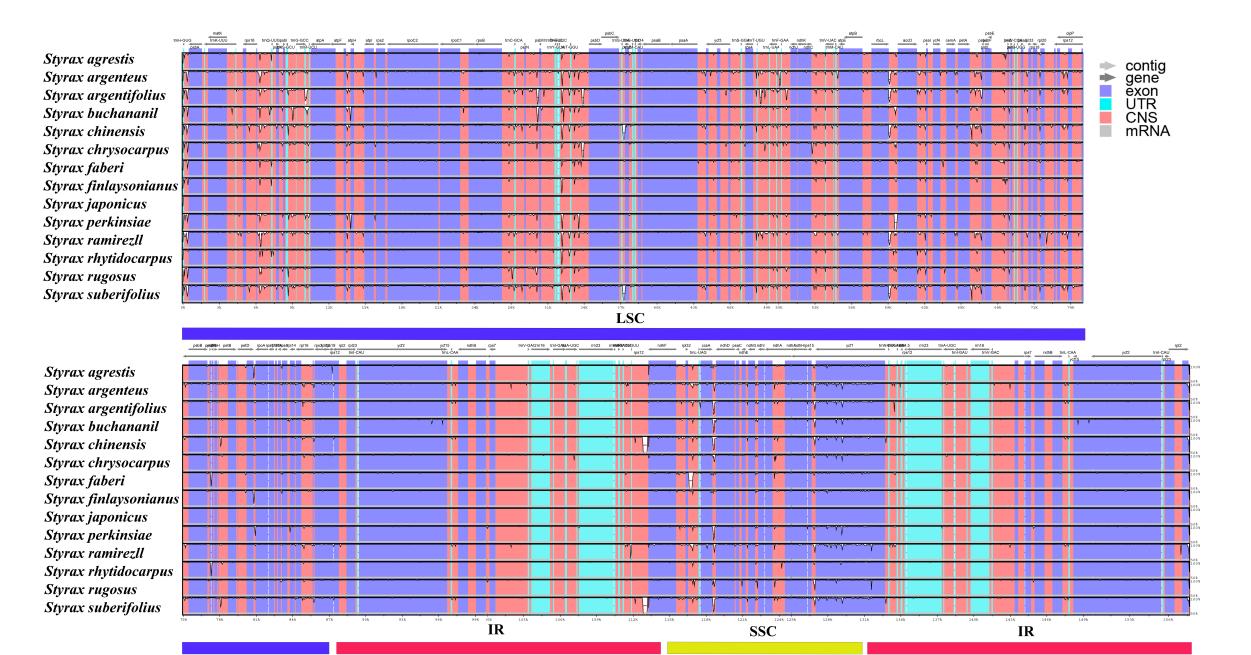


**Figure S2.** Variation level of of three varieties chloroplast genome sequences, the y-axis indicates the level of variation (between 50 and 100%) and the x-axis represents the coordinate in the chloroplast genome.

**Supplement Table**

**Table S1.** Characteristics of assembled chloroplast genome basic structure.

| **Species** | **Total size** | **GC content (%)** | **LSC size** | **IR size** | **SSC size** | **Protein coding genes** | **rRNA** | **tRNA** | **NCBI accession number** |
| --- | --- | --- | --- | --- | --- | --- | --- | --- | --- |
| ***S.argentifolius*** | 158015 bp | 37.0% | 87782 bp | 25945 bp | 18343 bp | 87 | 8 | 37 | PQ276582 |
| ***S.buchananii*** | 157979 bp | 37.0% | 87534 bp | 26086 bp | 18299 bp | 87 | 8 | 37 | PQ276583 |
| ***S.chrysocarpus*** | 157817 bp | 37.0% | 87420 bp | 26051 bp | 18295 bp | 87 | 8 | 37 | PQ276584 |
| ***S. finlaysonianus*** | 157929 bp | 37.0% | 87706 bp | 25971 bp | 18281 bp | 87 | 8 | 37 | PQ276585 |
| ***S.rhytidocarpus*** | 157997 bp | 36.9% | 87608 bp | 26045 bp | 18299 bp | 87 | 8 | 37 | PQ276586 |

**Table S2.** Lists of genomic genes for five species. Notes:Gene*: Gene with one introns; Gene**: Gene with two introns; #Gene: Pseudo gene; Gene(2): Number of copies of multi-copy genes

| **Category** | **Function** | ***S.argentifolius*** | ***S.buchananii*** | ***S.chrysocarpus*** | ***S. finlaysonianus*** | ***S.rhytidocarpus*** |
| --- | --- | --- | --- | --- | --- | --- |
| Photosynthesis | Subunits of photosystem I | *psaA,psaB,psaC,psaI,psaJ* | | | | |
|  | Subunits of photosystem II | *psbA,psbB,psbC,psbD,psbE,psbF,psbH,*  *psbI,psbJ,psbK,psbL,psbM,psbN,psbT* | | | | |
|  | Subunits of NADH  dehydrogenase | *ndhA*, ndhB*(2), ndhC,ndhD,ndhE,ndhF,ndhG,ndhH,ndhI,ndhJ,ndhK* | | | | |
|  | Subunits of cytochrome  b/f complex | *petA,petB*,petD*,petG,petL,petN* | | | | |
|  | Subunits of ATP synthase | *atpA,atpB,atpE,atpF*,atpH,atpI* | | | | |
|  | Large subunit of rubisco | *rbcL* | | | | |
|  | Subunits photochlorophyllide reductase | - | | | | |
| Self-replication | Proteins of large  ribosomal subunit | *rpl14,rpl16*,rpl2*(2),rpl20,rpl22,rpl23(2),rpl32,rpl33,rpl36* | | | | |
|  | Proteins of small  ribosomal subunit | *rps11,rps12**(2),rps14,rps15,rps16*,*  *rps18,rps19,rps2,rps3,rps4,rps7(2),rps8* | | | | |
|  | Subunits of RNA polymerase | *rpoA,rpoB,rpoC1*,rpoC2* | | | | |
|  | Ribosomal RNAs | *rrn16(2),rrn23(2),rrn4.5(2),rrn5(2)* | | | | |
|  | Transfer RNAs | *trnA-UGC*(2),trnC-GCA,trnD-GUC,trnE-UUC,trnF-GAA,*  *trnG-GCC*,trnG-UCC,trnH-GUG,trnI-CAU(2),trnI-GAU*(2),*  *trnK-UUU*,trnL-CAA(2),trnL-UAA*,trnL-UAG,trnM-CAU,*  *trnN-GUU(2),trnP-UGG,trnQ-UUG,trnR-ACG(2),trnR-UCU,*  *trnS-GCU,trnS-GGA,trnS-UGA,trnT-GGU,trnT-UGU,*  *trnV-GAC(2),trnV-UAC*,trnW-CCA,trnY-GUA,trnfM-CAU* | | | | |
| Other genes | Maturase | *matK* | | | | |
|  | Protease | *clpP*** | | | | |
|  | Envelope membrane protein | *cemA* | | | | |
|  | Acetyl-CoA carboxylase | *accD* | | | | |
|  | c-type cytochrome  synthesis gene | *ccsA* | | | | |
|  | Translation initiation factor | *infA* | | | | |
|  | other | - | | | | |
| Genes of unknown function | Conserved hypothetical  chloroplast ORF | *ycf1,lhbA,ycf1,ycf15(2),ycf2(2),ycf3**,ycf4* | | | | |
